# Verses

**Published:** 1906-07

**Authors:** 


### VERSES.

### HOT ENOUGH.

"Is it hot enough for you?"
They asked the ice man.
And when he answered "No,"
They told him where to go
And named a place repellant to a nice man.

---

### HOT BATH.

Don't sit down and sadly mumble,
Don't let others hear you grumble—
Take a good hot bath.
Do not, nursing anger, frown—it
Doesn't pay—go home and drown it
In a good hot bath.

—*S. E. Kiser.*

---

### OLD WOMAN DOCTOR.

There was once an old Woman Dr.
Whose patients' bad language oft shr.
Though she always would laugh
At their malice and chaugh,
And said that she cared not who knr.

—*Exchange.*

---

### HAPPINESS.

Happiness is like a crystal
Fair and exquisite and clear,
Broken in a million pieces,
Shattered, scattered, far and near.
Now and then, along life's pathway,
Lo! some shining fragments fall—
But there are so many pieces
No one ever finds them all.

—*Annuity Messenger.*

### ALL SORTS AND ALL SIZES.

I shall not pass this way again,
But far beyond earth's Where and When
May I look back along a road
Where on both sides good seed I sowed.
I shall not pass this way again,
May wisdom guide my tongue and pen,
And love be mine that so I may
Plant roses all along the way.
I shall not pass this way again;
May I be courteous to men;
Faithful to friends, true to my God,
A fragrance on the path I trod.

—*Newspaper.*

---

### THE NEGRO AND THE WATERMELON.

How wud a nigger do 'dout fruit,
  Long een de summer way down souf?
He'd srink up sho, dat's de troot,
  Ef sum kine ernurrer didn't hit 'e mouf.

De blackberry am er old stanby,
  De huckleberry am ernurrer old fren,
De strawberry? Hit cost too high.
  But nun of dese satterfacshun sen.

Peaches and pear am bofe purty good,
  An de ole fiel plums gits dar sum;
Canterlopes am brekfas food,
  But de watermelon does mek me hum.

Hits de king ob all dats sweet,—
  Hits so jucy an so fielin
Dat wen I stuffs on hits red meat,
  I cud go ter Heben, if de Lawd wus willin.

—*B. H. Teague.*

---

We are chloroforming grandpa—
  Don't you hear his feeble moan?
Grandpa is a nice old fellow
  And it's sad to have him groan—
Shall we take him out, my brothers,
Ere he dies beneath the lid?
No! we've talked with Dr. Osler,
  And he says it must be did.

